# Physical activity and osteosarcopenia in Korean adults aged 65 years and older: a national cross-sectional study using the KNHANES data

**DOI:** 10.1186/s12877-023-04121-8

**Published:** 2023-07-07

**Authors:** Byung Chan Lee, Kang Hee Cho, Chang-Won Moon

**Affiliations:** 1 Department of Physical Medicine and Rehabilitation, Chung-Ang University Hospital, Seoul, Korea; 2 Department of Rehabilitation Medicine, Chungnam National University College of Medicine, 266 Munhwa-ro, Jung-gu, Daejeon, 35015 Korea; 3 Department of Biomedical Institute, Chungnam National University, Daejeon, Korea

**Keywords:** Osteoporosis, Sarcopenia, Osteosarcopenia, Exercise, Strengthening exercise, Cross-sectional study

## Abstract

**Background:**

Osteosarcopenia is a syndrome characterized by the co-existence of osteoporosis and sarcopenia. This study aimed to examine the relationship between various types of physical activity and osteosarcopenia in community-dwelling Korean adults aged 65 years or older.

**Methods:**

This cross-sectional study used raw data from the fourth and fifth editions of the Korean National Health and Nutritional Survey Examination, conducted from 2008 to 2011. The researchers exclusively recruited participants aged 65 years or older for the study. These participants were categorized into four distinct groups based on their clinical factors, namely individuals without osteoporosis or sarcopenia, those with osteoporosis alone, those with sarcopenia alone, and individuals with osteosarcopenia. The International Physical Activity Short-Form was used to calculate the weekly time spent walking, moderate-intensity aerobic physical activity, and vigorous aerobic physical activity. Number of days in performing strengthening or stretching exercises were also surveyed. We used logistic regression analyses to examine the association between various physical activities and occurrence of osteosarcopenia.

**Results:**

A total of 1,342 participants (639 men and 703 women) were included in the analysis. No significant difference was observed in the amount and level of aerobic physical activity between the groups. The odds ratios below were based on participants without osteoporosis or sarcopenia as the reference category. The un-adjusted odds ratio of participants who performed stretching (male, 0.179, 95% CI 0.078–0.412; female 0.430, 95% CI 0.217–0.853) and strengthening exercises (male, 0.143, 95% CI 0.051–0.402; female, 0.044, 95% CI 0.006–0.342) at least twice per week was significantly lower in participants with osteosarcopenia compared to those without. In the adjusted analysis (adjusted by age, body mass index, house income, educational level, smoking habits, drinking status, and protein intake), only female patients in the osteosarcopenia group had a significantly lower adjusted odds ratio for performing strengthening exercise compared to female participants without osteoporosis or sarcopenia (0.062, 95% CI 0.007–0.538).

**Conclusions:**

After adjusting for confounding factors and protein intake, women aged 65 years and older who suffered osteosarcopenia had considerably lower odds ratio of performing strengthening exercises.

## Background

During the aging process, the loss of bone and muscle tissues occurs. Osteoporosis (OP) is defined as the decrement of bone mineral density (BMD) and having a T-score of less than − 2.5. This T-score signifies a comparison to the average BMD of a healthy young adult population, highlighting the extent of bone density loss in individuals with osteoporosis [1]. The worldwide prevalence of osteoporosis in the older population is approximately 21.7%, with females showing a prevalence of 35.3% and males showing a prevalence of 12.5% [2]. It is estimated that osteoporotic fractures can occur in about one-third of women and one-fifth of men after the age of 50 [3]. Similar to the decline in bone mass that occurs with aging, sarcopenia (SP) is a skeletal muscle disorder characterized by several key features. These include muscle weakness, loss of muscle mass, and a decline in physical performance [4]. The prevalence of sarcopenia in the community-dwelling population is reported to be between 1% and 29% [5]. Sarcopenia increases the risk of falls and associated fractures, causes disabilities affecting activities of daily living, and is associated with several conditions such as cardiac diseases, respiratory diseases, cognitive dysfunction, and potentially death [6].

Osteosarcopenia (OS) is a syndrome characterized by the co-existence of both OP and SP [7]. OS prevalence is rarely reported due to its novelty as a condition. However, in a study conducted on 1,003 patients who had suffered hip fractures in Korea, it was found that 61% of the male participants and 26% of the female participants exhibited sarcopenia [8]. Moreover, a study conducted on Chinese individuals aged ≥ 80 years reported that the prevalence of osteosarcopenia was 10.4% in males and 15.1% in females [9]. OS cases are predicted to increase in the future with the aging population, increasing the risk of fragility fractures and increasing socioeconomic costs, morbidity, and mortality [10–12].

Muscles and bones are interconnected organs that are known to share the mechanical effect of muscle loading on bone function [13]. Decreased physical activity levels are associated with aging and have a detrimental impact on both muscle and bone health, as seen in older individuals who spend up to 80% of their waking hours inactive [14]. Several non-pharmacological guidelines for OP [15, 16] and SP [17–19] patients have emerged in the past decade and focused on the value of resistance exercise and high impact activity.

Accordingly, promoting the augmentation of peak bone and muscle mass during middle age, as well as averting the decline of bone and muscle mass in later stages of life, is considered the appropriate non-pharmacological management for OS patients. Furthermore, comprehending the patterns of physical inactivity in OS patients can serve as a crucial step in identifying modifiable risk factors and developing effective management plans. Understanding the relationship between physical activity levels and OS in patients can be the key to effectively addressing this detrimental consequence of the aging process.

This study aimed to investigate the association between various types of physical activities and patients with OS in a nation-wide representative sample of community-dwelling older adults in Korea.

## Methods

### Study participants

This cross-sectional study used raw data from the fourth and fifth editions of the Korean National Health and Nutritional Survey Examination (KNHANES), conducted from 2008 to 2011 by the Korea Centers for Disease Control and Prevention (KCDC), Ministry of Health and Welfare. The KNHANES study is a nationwide cross-sectional survey conducted annually using a stratified, multistage clustered probability sampling design established in 1998 [20].

The KNHANES includes a health interview, nutritional survey, and health-related exam including dual-energy X-ray absorptiometry (DEXA) among the participants. DEXA was performed in subgroups of participants which consisted of men and post-menopausal women aged 50 or over from July 2008 to May 2011.

The total population included in the KNHANES from 2008 to 2011 was 37,753. A total of 6,370 participants (2,647 men and 3,723 women) are ≥ 65 years of age. In this study, we only included 3,867 participants (1,687 men and 2,180 women) who completed DEXA. Patients with missing DEXA data were excluded (130 men and 303 women). Individuals who did not respond to physical activity (918 men and 1,174 women) were also excluded. A total of 1,342 participants (639 men and 703 women) were included in the final analysis. The researchers then categorized these participants into four distinct groups based on their clinical factors, namely individuals without osteoporosis or sarcopenia, those with osteoporosis alone, those with sarcopenia alone, and individuals with osteosarcopenia. This study was approved by the Institutional Review Board of the Korean National Health and Nutritional Survey Examination and Korean National Health and Nutritional Survey Examination (2008-04EXP-01-C, 2009-01CON-03-2 C, 2010-02CON-21-C, 2011-02CON-06-C). All participants provided written informed consent.

### Evaluation of osteosarcopenia factors and diagnosis

OP and SP were evaluated using DEXA (Hologic®; Hologic Discovery, Hologic, Bedford, MA, USA). Low bone mass of the total hip, femoral neck, and/or lumbar spine was diagnosed as osteoporosis if the T-score was less than − 2.5 [21]. SP was assessed using the skeletal muscle mass index (SMI) calculated from appendicular skeletal muscle (ASM) mass (kg) divided by the height squared (m^2^) [22]. Authors used standards from the Asian Working Group for Sarcopenia (AWGS) published in 2019, and sarcopenia was diagnosed by DEXA when the SMI was less than 7.0 kg/m^2^ in men and less than 5.4 kg/m^2^ in women [18]. Participants were classified into OP groups if they met the criteria for the diagnosis of OP and did not meet the criteria for SP, and vice versa into SP groups. Participants who met both criteria for OP and SP were classified as having OS.

### Anthropometric and laboratory measurements

The baseline demographic factors of the participants were obtained through health interviews and examinations. The units of measurement for body weight and height were kg and cm, respectively. Blood samples were collected from the antecubital veins of participants aged 10 years and older, were stored at 4 °C immediately, and were analyzed within 24 h of sampling. Blood samples were analyzed to estimate the prevalence of diabetes, dyslipidemia, and infectious diseases, as well as to gather information on exposure to tobacco smoke and heavy metals, kidney function, and thyroid function as part of the KNHANES study. The detailed data resource profile and the methods and devices used in measurements are described elsewhere [20].

### Physical activity

The KNHANES study used the Korean version of International Physical Activity Short-Form (IPAQ-SF) to evaluate the physical activity of the participants [23]. The IPAQ-SF was used to calculate the weekly time spent walking, doing moderate-intensity aerobic physical activity (PA), and doing vigorous aerobic PA. The total amount of aerobic PA was calculated by adding the minutes spent in moderate and doubling the minutes spent in vigorous aerobic PA each week. Walking was not included in the total minutes of aerobic PA because it was considered low-intensity aerobic PA. Using the American College of Sports Medicine (ACSM) guidelines, the total amount of aerobic PA was categorized into three groups: completely inactive (0 min of total aerobic PA), insufficiently active (1–149 min of moderate to vigorous aerobic PA per week), and physically active (150 min or more of moderate to vigorous aerobic PA per week) [24]. Participants were asked to report the number of days they engaged in walking, stretching, and strengthening physical activities during the previous week. Based on their responses, the data were categorized as follows. The active walking was defined as walking for 5 or more days per week, with each session lasting at least 30 min. As per the stretching and strengthening exercises, the ACSM guidelines for older adults specified a minimum of 2 or more days per week. Participants who met this criterion were classified as actively engaging in stretching or strengthening exercises.

### Nutritional survey

Participants were asked about their average amount of dietary intake using a 1-day 24-h recall method. In-person interviews were conducted by trained survey staff to investigate the quantity and types of food intake consumed the day before the survey, as well as the quantity and type of ingredients by food item. Two-dimensional food containers and food models, measuring spoons and cups, thickness sticks, 30-cm rulers, and tape measures were employed to ensure the survey’s accuracy. Subsequently, the total daily energy (kcal), protein (g), and calcium (mg) consumption were computed from the survey. The detailed data resource profile is described elsewhere [20].

### Covariates

The study covariates included smoking status, drinking status, socioeconomic factors, protein intake, and BMI as important determinants of metabolic risk factors and/or comorbidities. The average amount and frequency of alcohol consumption for the month before the interview was recorded. There were three categories of alcohol consumption status: non-drinking, low-risk drinking, and high-risk drinking. High-risk drinkers were defined as those consuming more than seven drinks at once and more than two days per week. The participants were divided into three groups: current smokers, ex-smokers, and non-smokers. The socioeconomic variables were household income and educational level. Family income was classified by quartile as low, moderate-to-low, moderate-to-high, and high. Additionally, education levels were divided into four groups: elementary school or less (≤ 6 years), middle school (7–9 years), high school (10–12 years), and college and above (≥ 13 years).

### Statistical analysis

Due to the nature of the KNHANES study using stratified cluster sampling methods, data were weighted based on the year of the survey and the stratification and cluster variables by creating a complex sample analysis plan file based on the sample weights provided. For continuous data, weighted means and standard errors were used. For categorical data, frequencies with weighted percentages were used. Analysis of variance (ANOVA) and chi-square tests were used to examine differences between the four groups of the study. Bonferroni correction was used to adjust the P-values to evaluate differences between the groups in the post-hoc analysis. To ascertain the association between PA (aerobic exercise, walking activity, stretching, and strengthening exercises) and each group, complex sample logistic regression analyses [25] were performed using both unadjusted and multivariable-adjusted analyses. For this purpose, the data stratified by sex were adjusted for age, BMI, educational attainment, familial income, drinking and smoking habits, and protein intake. All analyses were conducted using IBM SPSS 23.0. All tests of statistical significance were two-sided, with a *P* Value of 0.05.

## Results

### Clinical and socioeconomic status among OS phenotypes

Table 1 shows the general characteristics of the study population, which comprised 639 men (mean age: 70.98 years) and 703 women (mean age: 71.61 years). The prevalence of OP, SP, and OS was significantly different between sexes (*P* < 0.001). The prevalence of osteoporosis was low in males (neither OP nor SP: 63.4%, OP: 2.1%, SP: 27.5%, and OS: 7.0%), whereas that of sarcopenia was low in females (neither OP nor SP: 39.7%, OP: 47.0%, SP: 3.3%, and OS: 9.9%) (Table 1).

**Table 1:** General characteristics of the included population

	Men (*n* = 639)	Women (*n* = 703)	*P*-value
Age	70.98 ± 0.19	71.61 ± 0.23	0.034
Height (cm)	165.27 ± 0.27	151.26 ± 0.25	< 0.001
Weight (kg)	63.70 ± 0.53	55.82 ± 0.43	< 0.001
BMI	23.26 ± 0.16	24.33 ± 0.15	< 0.001
Nutrient intake			
Total kcal	1967.69 ± 37.18	1459.13 ± 21.77	< 0.001
Protein intake (g)	66.52 ± 1.57	46.53 ± 1.01	< 0.001
BMD			
Total femur BMD	0.90 ± 0.006	0.72 ± 0.005	< 0.001
Femoral neck BMD	0.72 ± 0.006	0.57 ± 0.004	< 0.001
Lumbar spine BMD	0.94 ± 0.008	0.74 ± 0.006	< 0.001
Total Femur BMD(T-score)	-0.32 ± 0.047	-1.13 ± 0.041	< 0.001
Lumbar spine BMD(T-score)	-0.73 ± 0.064	-2.32 ± 0.049	< 0.001
Femoral neck BMD (T-score)	-1.03 ± 0.047	-2.18 ± 0.037	< 0.001
SMI (kg/m^2^)^a^	7.27 ± 0.04	6.02 ± 0.03	< 0.001
Group			< 0.001
Neither OP nor SP	395 (63.4%)	292 (39.7%)	
OP	15 (2.1%)	314 (47.0%)	
SP	187 (27.5%)	28 (3.3%)	
OS	42 (7.0%)	69 (9.9%)	

Weighted means and standard errors were used for continuous data, while frequencies with weighted percentages were used for categorical data. BMI, Body mass index; BMD, Bone mineral density; ASM, Appendicular skeletal muscle; SMI, Skeletal muscle index; OP, Osteoporosis; SP, Sarcopenia; OS, Osteosarcopenia

^a^SMI was calculated by dividing appendicular skeletal muscle by the square of height

Among the male subjects, the OP, SP, and OS groups had a higher mean age than that of the group with neither OP nor SP (Table 2). Height was lower in the male OP and OS groups, while weight and BMI were lower in the male SP and OS groups (Table 2). The total caloric and protein intakes were also remarkably lower in the male OP, SP, and OS groups than in the neither OP nor SP group (Table 2). Compared with males in the neither OP nor SP group, the OS group comprised a markedly lower percentage of male subjects who had completed their college or high school education and had a high household income level (Table 2).

**Table 2:** General characteristics and physical activity of the male participants according to the osteosarcopenia phenotype

Men (S.E)	Neither OP nor SP(*N* = 395)	OP (*N* = 15)	SP (*N* = 187)	OS (*N* = 42)	*P*-value
Age	69.93 ± 0.23	74.00 ± 1.44^a^	72.51 ± 0.36^a^	73.60 ± 0.78^a^	< 0.001
Height (cm)	166.02 ± 0.32	161.21 ± 0.98^a^	164.72 ± 0.45	161.87 ± 1.30^a^	< 0.001
Weight (kg)	67.65 ± 0.53	64.22 ± 2.00	57.66 ± 0.70^a^	51.47 ± 1.23^a^	< 0.001
BMI	24.51 ± 0.17	24.66 ± 0.64	21.20 ± 0.20^a^	19.61 ± 0.33^a^	< 0.001
Nutrient intake					
Total kcal	2086.00 ± 47.00	1772.03 ± 111.68^a^	1807.01 ± 60.42^a^	1592.13 ± 84.95^a^	< 0.001
Protein intake (g)	70.42 ± 1.86	58.82 ± 4.60^a^	62.28 ± 2.91^a^	50.17 ± 4.54^a^	< 0.001
Family income					0.007
Low	143 (34.2%)	10 (76.7%)	83 (45.7%)	26 (55.1%)	
Moderate-low	126 (33.7%)	2 (15.1%)	52 (27.5%)	12 (37.7%)	
Moderate-high	67 (17.6%)	0 (0.0%)	31 (16.9%)	2 (3.0%)	
High	54 (14.5%)	3 (8.2%)	19 (9.9%)	2 (4.2%)	
Education					0.013
<Elementary	169 (45.7%)	8 (70.3%)	81 (41.5%)	29 (70.2%)	
Middle school	74 (17.4%)	3 (14.4%)	31 (18.9%)	8 (21.3%)	
High school	90 (21.6%)	4 (15.3%)	38 (17.7%)	2 (3.5%)	
>College	62 (15.3%)	0 (0.0%)	36 (21.9%)	3 (5.0%)	
Smoking Status					0.400
None	70 (24.5%)	2 (21.0%)	37 (27.4%)	7 (18.4%)	
Ex-smoker	136 (46.7%)	2 (19.4%)	55 (40.4%)	13 (38.9%)	
Current smoker	85 (28.8%)	5 (59.6%)	41 (32.2%)	11 (42.6%)	
Alcohol Drinking					0.511
None	104 (27.4%)	3 (20.7%)	55 (29.4%)	16 (41.1%)	
Low risk drinking	249 (61.2%)	12 (79.3%)	111 (59.2%)	22 (45.9%)	
High risk drinking	40 (11.4%)	0 (0.0%)	21 (11.4%)	4 (13.0%)	
PA (minute/week)	79.17 ± 7.14	57.47 ± 24.46	87.10 ± 13.56	70.84 ± 22.09	0.744
PA (group)					0.868
Completely inactive	239 (59.7%)	10 (63.6%)	119 (59.6%)	28 (67.6%)	
Insufficiently active	63 (16.7%)	2 (12.1%)	23 (15.4%)	3 (7.0%)	
Physically active	93 (23.6%)	3 (24.4%)	45 (25.0%)	11 (25.4%)	
Walking	232 (56.6%)	5 (46.4%)	111 (57.0%)	24 (48.7%)	0.743
Stretching exercise	206 (54.2%)	8 (55.2%)	84 (49.4%)	9 (17.5%)	< 0.001
Strengthening exercise	153 (40.1%)	4 (26.9%)	58 (31.8%)	5 (8.7%)	0.001

Weighted means and standard errors were used for continuous data, while frequencies with weighted percentages were used for categorical data. The p-values reflect overall differences in characteristics among the four osteosarcopenia phenotypes. BMI, Body mass index; OP, Osteoporosis; SP, Sarcopenia; OS, Osteosarcopenia

^a^Statistically significant difference from the neither OP nor SP group after post-hoc analysis using Bonferroni correction (*P-*value < 0.0167)

Among the female subjects, OP and OS groups were markedly older than those in the neither OP nor SP group (Table 3). Height was lower in the female OP and OS groups, while weight and BMI were lower in the female OP, SP, and OS groups as compared to the neither OP nor SP group. The total caloric intake was considerably lower in the OS group; total protein intake was lower in the OP group (Table 3). Compared with the neither OP nor SP group, the OS group comprised a markedly lower percentage of female subjects who had completed their college or high school education; however, there were no significant differences between household income levels in females (Table 3).

**Table 3:** General characteristics, nutrition, and socioeconomic status of the female participants according to the osteosarcopenia phenotype

Women (S.E)	Neither OP nor SP (*N* = 292)	OP (*N* = 314)	SP (N = 28)	OS (N = 69)	*P*-value
Age	70.22 ± 0.29	72.52 ± 0.34^a^	71.67 ± 0.89	72.86 ± 0.69^a^	< 0.001
Height (cm)	153.03 ± 0.38	149.99 ± 0.30^a^	152.12 ± 0.95	149.88 ± 0.96^a^	< 0.001
Weight (kg)	60.56 ± 0.580	53.81 ± 0.54^a^	54.05 ± 1.43^a^	46.94 ± 0.88^a^	< 0.001
BMI	25.82 ± 0.21	23.88 ± 0.22^a^	23.33 ± 0.50^a^	20.84 ± 0.27^a^	< 0.001
Nutrient intake					
Total kcal	1516.14 ± 34.20	1452.36 ± 33.53	1403.05 ± 124.16	1295.56 ± 59.63^a^	0.014
Protein intake (g)	48.72 ± 1.54	45.70 ± 1.57^a^	49.29 ± 5.69	41.25 ± 2.75	0.107
Family income					0.161
Low	155 (50.7%)	193 (58.2%)	15 (58.9%)	36 (54.0%)	
Moderate-low	72 (25.9%)	62 (21.7%)	3 (10.4%)	25 (36.0%)	
Moderate-high	33 (13.1%)	32 (11.4%)	4 (10.7%)	5 (4.9%)	
High	30 (10.3%)	23 (8.7%)	6 (20.1%)	3 (5.1%)	
Education					0.032
<Elementary	237 (79.4%)	284 (91.0%)	20 (78.9%)	61 (88.4%)	
Middle school	25 (10.5%)	14 (4.1%)	5 (13.4%)	3 (3.3%)	
High school	24 (8.2%)	9 (3.5%)	3 (7.7%)	5 (8.3%)	
>College	6 (1.9%)	6 (1.5%)	0 (0.0%)	0 (0.0%)	
Smoking Status					0.011
None	276 (96.4%)	281 (90.7%)	26 (97.5%)	59 (83.8%)	
Ex-smoker	7 (2.1%)	9 (2.5%)	1 (2.5%)	2 (3.2%)	
Current smoker	7 (1.5%)	18 (6.8%)	0 (0.0%)	8 (13.0%)	
Alcohol Drinking					0.276
None	180 (59.9%)	219 (71.1%)	21 (70.7%)	49 (66.9%)	
Low risk drinking	111 (39.5%)	93 (28.0%)	7 (29.3%)	20 (33.1%)	
High risk drinking	1 (0.6%)	2 (0.9%)	0 (0.0%)	0 (0.0%)	
PA (minute/week)	73.54 ± 6.85	79.41 ± 7.29	45.40 ± 18.76	74.20 ± 15.49	0.404
PA (group)					0.849
Completely inactive	171 (55.6%)	171 (53.4%)	18 (68.5%)	40 (58.8%)	
Insufficiently active	59 (20.9%)	72 (23.3%)	7 (20.1%)	13 (19.8%)	
Physically active	62 (23.5%)	71 (23.3%)	3 (11.5%)	16 (21.4%)	
Walking	139 (49.3%)	138 (46.0%)	17 (66.3%)	35 (46.9%)	0.378
Stretching exercise	123 (45.0%)	90 (28.2%)	15 (47.9%)	19 (26.0%)	< 0.001
Strengthening exercise	37 (14.1%)	25 (6.3%)	3 (12.5%)	1 (0.7%)	0.001

Weighted means and standard errors were used for continuous data, while frequencies with weighted percentages were used for categorical data. The p-values reflect overall differences in characteristics among the four osteosarcopenia phenotypes. BMI, Body mass index; OP, Osteoporosis; SP, Sarcopenia; OS, Osteosarcopenia

^a^Statistically significant difference from the neither OP nor SP group after post-hoc analysis using Bonferroni correction (*P*-value < 0.0167)

### Lifestyle Habitual and Physical Activity between OS Phenotypes

Smoking and drinking statuses were not significantly different between the OS phenotypes in males (Table 2). However, smoking status was extensively different between the OS phenotypes in females (Table 3). The amount of aerobic physical activity was not significantly different between the OS phenotypes in both sexes, and there was no significant difference in the level of physical activity (completely inactive, insufficiently active, and physically active) between the OS phenotypes in both sexes (Tables 2 and 3). However, the proportion of participants who performed stretching and strengthening exercises at least two days per week was statistically significant (*P* < 0.05) and lower in the OS groups of both sexes (Tables 2 and 3).

The odds ratios presented below were all based on the reference category of participants who did not have osteoporosis or sarcopenia. In complex sample logistic regression analysis, the odds ratios of being physically active in aerobic exercise or walking activity in the OS group were not statistically significant (men: physically active, 1.099, 95% CI 0.465–2.599 and walking activity, 0.727, 95% CI 0.349–1.514, Table 4; women: physically active, 0.886, 95% CI 0.428–1.834 and walking activity, 0.907, 95% CI 0.484–1.702, Table 5). However, the odds ratios of participants in the OS group who did stretching exercise more than 2 days per week were significantly less than those in the neither OP nor SP group in both sexes (men: 0.179, 95% CI 0.078–0.412, Table 4; women: 0.430, 95% CI 0.217–0.853, Table 5). The same trend was observed in participants of the OS groups doing strengthening exercise (men: 0.143, 95% CI 0.051–0.402, Table 4; women: 0.044, 95% CI 0.006–0.342, Table 5).

**Fig. 1:**
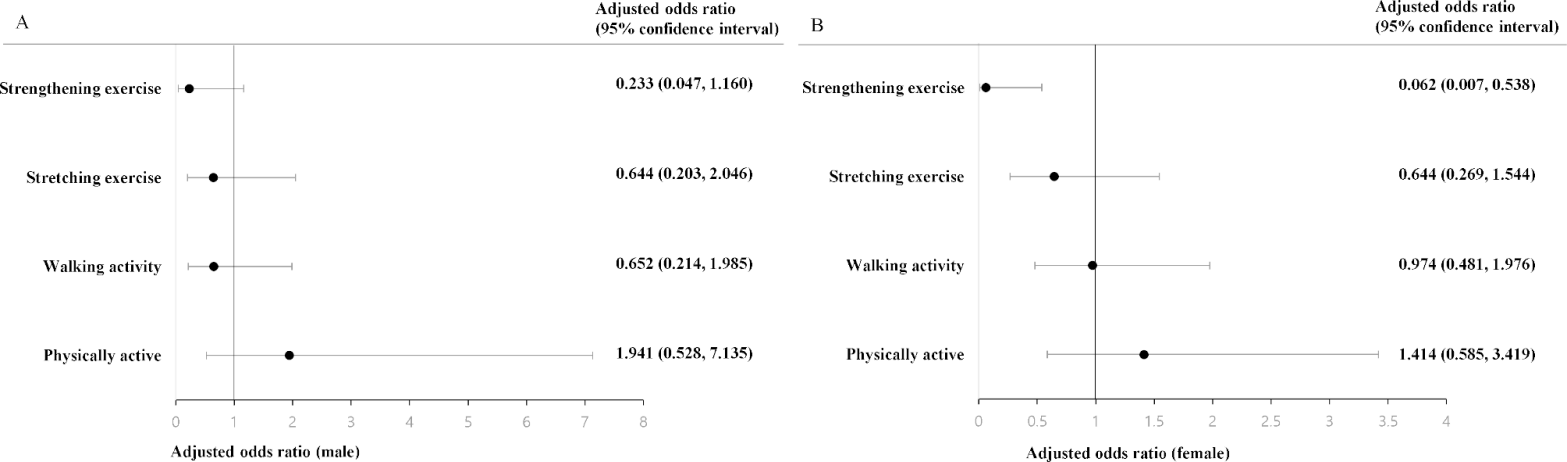
Odds ratios of various physical activities in OS patients compared with those in the neither OP nor SP population in men (**A**) and women (**B**) in adjusted logistic regression analysis. Only female OS patients had a significantly lower odds ratio to attain at least two days of strengthening exercise per week than female participants in the neither OP nor SP group (0.062, 95% CI 0.007–0.538, P = 0.048)

However, the statistical significances diminished in male patients (Table 4) when adjusted by age, body mass index (BMI), household income, educational attainment, smoking and drinking, and protein intake. Moreover, only female OS patients had a significantly lower odds ratio to attain at least two days of strengthening exercise per week than those in the neither OP nor SP group (0.062, 95% CI 0.007–0.538) (Fig. 1; Table 5). In unadjusted analysis, the odds ratios in OP female participants who engaged in stretching and strengthening exercises were remarkably lower compared to those in the neither OP nor SP group. However, in adjusted analysis, only the odds ratio in stretching exercises remained significantly lower compared to in the neither OP nor SP group (0.606, 95% CI 0.392–0.936, Table 5).

**Table 4:** Results of the regression analysis of physical activity in male participants according to the osteosarcopenia phenotype

Men	Neither OP nor SP	OP	SP	OS
Crude				
Physically active^a^	1.00 (reference)	1.040 (0.231, 4.686)	1.078 (0.681, 1.704)	1.099 (0.465, 2.599)
Walking activity^b^	1.00 (reference)	0.663 (0.188, 2.345)	1.013 (0.679, 1.512)	0.727 (0.349, 1.514)
Stretching exercise^c^	1.00 (reference)	1.044 (0.326, 3.340)	0.827 (0.549, 1.245)	**0.179 (0.078, 0.412)**
Strengthening exercise^c^	1.00 (reference)	0.549 (0.146, 2.066)	0.698 (0.439, 1.108)	**0.143 (0.051, 0.402)**
Adjusted^d^				
Physically active^a^	1.00 (reference)	1.112 (0.215, 5.762)	1.308 (0.614, 2.785)	1.941 (0.528, 7.135)
Walking activity^b^	1.00 (reference)	0.890 (0.206, 3.847)	0.710 (0.382, 1.322)	0.652 (0.214, 1.985)
Stretching exercise^c^	1.00 (reference)	0.380 (0.076, 1.909)	0.998 (0.506, 1.967)	0.644 (0.203, 2.046)
Strengthening exercise^c^	1.00 (reference)	0.194 (0.022, 1.741)	1.031 (0.496, 2.141)	0.233 (0.047, 1.160)

OP, Osteoporosis; SP, Sarcopenia; OS, Osteosarcopenia

^a^Physically active was defined as 150 min or more of moderate to vigorous aerobic PA per week

^b^Walking activity was defined as 5 or more days of walking at least 30 min per day

^c^Strengthening or stretching exercises were defined as at least 2 or more days of exercise per week

^d^adjusted by age, body mass index, house income, educational level, smoking habits, drinking status, and protein intake

**Table 5:** Results of the regression analysis of physical activity in female participants according to the osteosarcopenia phenotype

Women	Neither OP nor SP	OP	SP	OS
Crude				
Physically active^a^	1.00 (reference)	0.990 (0.644, 1.520)	0.421 (0.117, 1.512)	0.886 (0.428, 1.834)
Walking activity^b^	1.00 (reference)	0.876 (0.597, 1.286)	2.026 (0.867, 4.734)	0.907 (0.484, 1.702)
Stretching exercise^c^	1.00 (reference)	**0.480 (0.324, 0.711)**	1.124 (0.483, 2.614)	**0.430 (0.217, 0.853)**
Strengthening exercise^c^	1.00 (reference)	**0.411 (0.216, 0.782)**	0.869 (0.208, 3.632)	**0.044 (0.006, 0.342)**
Adjusted^d^				
Physically active^a^	1.00 (reference)	1.170 (0.717, 1.910)	0.568 (0.145, 2.227)	1.414 (0.585, 3.419)
Walking activity^b^	1.00 (reference)	0.955 (0.632, 1.444)	2.433 (0.998, 5.936)	0.974 (0.481, 1.976)
Stretching exercise^c^	1.00 (reference)	**0.606 (0.392, 0.936)**	1.363 (0.572, 3.243)	0.644 (0.269, 1.544)
Strengthening exercise^c^	1.00 (reference)	0.576 (0.272, 1.220)	1.193 (0.228, 6.227)	**0.062 (0.007, 0.538)**

OP, Osteoporosis; SP, Sarcopenia; OS, Osteosarcopenia

^a^Physically active was defined as 150 min or more of moderate to vigorous aerobic PA per week

^b^Walking activity was defined as 5 or more days of walking at least 30 min per day

^c^Strengthening or stretching exercises were defined as at least 2 or more days of exercise per week

^d^Adjusted by age, body mass index, house income, educational level, smoking habits, drinking status, and protein intake

## Discussion

This cross-sectional revealed remarkably decreased odds ratios for meeting the recommended physical activity levels in stretching and strengthening exercises among male patients with OS and female patients with OP and OS. However, after adjusting for confounding factors, the statistical significance was diminished in male OS patients. Female participants with OS exhibited a lower odds ratio for engaging in strengthening exercises compared to those with other OS-associated phenotypes. Additionally, female patients with OP exhibited lower odds ratios for engaging in stretching exercises. The authors clarify that the odds ratios presented in this study are based on the reference category of participants who did not have osteoporosis or sarcopenia. In the present study, OS was extensively associated with age, BMI, family income, and nutritional status in the older Korean population. To our knowledge, this is the first study to evaluate physical activity levels between the phenotypes of OS in an older population.

The multifactorial pathophysiology of osteosarcopenia consists of mechanical, biochemical, genetic, and lifestyle factors. Decreased physical activity levels associated with aging have a profound impact on both bone and muscle health [14]. This lack of physical activity in the elderly population could potentially result in a loss of mechanical loading, leading to muscle and bone loss and ultimately increasing the risk of fractures [16, 26, 27]. OS has been shown to be associated with depression, malnutrition, peptic ulcer disease, inflammatory arthritis, and reduced mobility [12]. Moreover, populations with OS are at a higher risk of falls and fractures than those with OP or SP alone [9, 12]. Therefore, it is important to encourage physical activity in vulnerable populations and to provide an appropriate exercise prescription for patients affected with OS.

This cross-sectional study showed that in the Korean older population, patients with osteosarcopenia spent a similar amount of time performing walking or aerobic exercise activities compared with healthy older patients. To our knowledge, walking or aerobic exercise habits in patients with osteosarcopenia have not been reported. Guidelines recommend physical activity for older adults and highlight the importance of achieving 150 min of moderate-to-vigorous-intensity physical activity per week [24, 28]. Although aerobic exercises have several definite benefits for older adults, older adults tend to have difficulty performing moderate and vigorous physical activity. Individuals between the ages of 70 and 79 spend approximately 9.6 h of their awake time engaging in sedentary activities, and less than 10–15% of older persons fulfill the recommended minimum of > 150 min per week of moderate-intensity activity [29]. Although we did not find a significant difference in the association between aerobic exercise in the neither OP nor SP population versus the OS-associated group in this study, it is important to note that the cross-sectional study did not measure the aerobic exercise activity of the participants during their middle-aged years, due to the nature of the study design. These outcomes suggest that osteosarcopenia had less of an impact on reducing the self-reported amount of aerobic exercise in older adults in Korea.

A systematic review of the non-pharmacological interventions in OS was recently published in 2021 and concluded that in older persons with osteosarcopenia, chronic resistance exercises are safe and effective at increasing or maintaining BMD and gaining muscle mass and quality [30]. In this cross-sectional study, although limited to women, a statistically significant (*P* < 0.05) decrease in the frequency of strengthening exercises was observed in patients with OS. It is unclear whether the decreased amount of strengthening exercises is the cause or consequence of OS. However, considering a previous report that described how exercise behaviors during middle age are linked to increased muscle strength and reduced prevalence of sarcopenia in older adults [31], sedentary habits could be a possible explanation for the development of osteosarcopenia in older Korean women.

Resistance training plays an important role in improving the physical performance of adults and promotes healthy aging. Resistance training and whole-body vibration exercises were linked to increased physical performance compared to standard care in a meta-analysis conducted in 2018 [32]. Although resistance exercise did not show a robust increase in lean body mass in the older population in this study, it is well known that it increases muscle strength and physical performance in sarcopenia [33–35] and osteoporosis patients [15, 36]. In contrast, endurance or aerobic exercise does not improve muscle strength or physical performance in older people [32].

Some arguments suggest that muscle strength alone may not be sufficient for performing functional activities. In addition to muscle strength, neuromuscular coordination plays a crucial role in movement velocity and can contribute to the improvement of functional movements [37]. Therefore, functional exercise to improve the limitations of daily activities should be considered rather than training programs that primarily focus on increasing isolated muscle strength [38]. Current research on osteosarcopenia focuses on the outcomes of BMD, muscle mass and quality, and physical functions [30]. However, osteosarcopenia is also associated with social frailty [39, 40]. Therefore, multicomponent interventions should be considered to impact different aspects of physical, psychosocial, or cognitive function in the older and osteosarcopenia patients [41].

This study had few limitations. First, the amount and intensity of physical activity were assessed based on self-reported measures following the guidelines provided by the IPAQ-SF questionnaire, which includes reporting the physical activity amount for both moderate and vigorous intensity exercises. However, it is important to note that the calculation of the total amount of physical activity was not originally included in the study design but was rather performed by the researchers for further analysis. Second, data from a cross-sectional study were used in this study. Therefore, a causal relationship could not be determined owing to the nature of the study design. Third, this study analyzed data based on the KNHANES 2008–2011 survey. The availability of DEXA scans during the 2008–2011 period allowed for analysis. However, it should be noted that measures of muscle strength, such as grip strength, were only included in the KNHANES study starting from 2014. As a result, the groups categorized as sarcopenia in this study, when applying AWGS criteria, only pertain to low muscle mass and cannot be precisely classified as sarcopenia. Therefore, it is important to acknowledge the potential presence of bias in these results. Fourth, when classifying the subjects into OS-related groups, the final sample size of the subjects was small in the male osteoporosis and female sarcopenia groups. However, the direct comparison of various physical activities between the neither OP nor SP and OS groups is a strength of this study. Furthermore, this study utilized data at the national level, which suggests that our conclusions could be extended to older Korean individuals.

## Conclusions

In conclusion, various physical activities showed similar tendencies in the OS-associated groups in both sexes. The OS group tended to spend less time performing stretching and strengthening exercises. In women aged ≥ 65 years, a decreased frequency of performing strengthening exercises was associated with a higher risk of OS after adjusting for several confounding factors and protein intake. These results may provide baseline data for establishing non-pharmacological guidelines for the management and prevention of OS. Further studies are warranted to clarify the causal relationships between OS and various physical activities.

## Data Availability

The datasets generated and/or analyzed during the current study are available in the Korea National Health & Nutrition Examination Survey repository, https://knhanes.kdca.go.kr/.

## List of abbreviations

ACSM: American College of Sports Medicine
ANOVA: Analysis of Variance
ASM: appendicular skeletal muscle
AWGS: Asian Working Group for Sarcopenia
BMI: Body Mass Index
DEXA: dual-energy X-ray absorptiometry
IPAQ-SF: International Physical Activity Short-From
KCDC: Korea Centers for Disease Control and Prevention
KNHANES: Korean National Health and Nutritional Survey Examination
OP: Osteoporosis
OS: Osteosarcopenia
PA: Physical Activity
SMI: skeletal muscle index
SP: Sarcopenia
